# Sexual Cyberaddiction and Attachment Style among Medical Students

**DOI:** 10.1192/j.eurpsy.2026.10507

**Published:** 2026-07-31

**Authors:** W. Ben Flah, S. Kolsi, I. Ghazzani, A. Kamoun

**Affiliations:** 1 Psychiatry, Faculty of medicine of Tunis, Tunis; 2 Psychiatry, Faculty of medicine of Sfax, Sfax, Tunisia

## Abstract

**Introduction:**

The rise of the Internet has facilitated new online sexual practices, exposing some users to the risk of problematic use. Data on this topic are scarce in Tunisia, particularly regarding the relationship with attachment style.

**Objectives:**

This study aimed to assess sexual cyberaddiction among medical students and examine the influence of attachment style.

**Methods:**

A cross-sectional descriptive and analytical study was conducted with 174 medical students. Online sexual practices were assessed using the French version of the s-IAT-sex, and attachment style was evaluated using the ECR-12. Sociodemographic, psychiatric, behavioral, and sexual data were collected via a self-administered e-questionnaire. Correlation and multivariate analyses were performed.

**Results:**

Our sample comprised young adults with a mean age of 25 years (SD = 3.6, range 18–34), predominantly female (74.7%; sex ratio M/F = 0.34). Most participants were externs (49.4%) or residents (42%), belonged to the middle socioeconomic class (81%), and identified as heterosexual (90.8%). Nearly half were single (49.4%), while one-third were in a relationship (35.6%). A history of somatic conditions was reported by 21.3% of participants, and psychiatric disorders by 20.7%, mainly mood (10.9%) and anxiety disorders (7.5%). Regular substance use was infrequent:(smoking 16.7%, alcohol 4%, cannabis 2.3%).

The mean s-IAT-sex score was 18 ± 8.5, reflecting generally non-problematic use. According to the ECR-12 scale, the most common attachment style was secure (48.3%), whereas fearful attachment was the least represented (3.5%). Kruskal-Wallis analysis showed significant differences in s-IAT scores across attachment styles, with the highest scores observed in individuals with fearful attachment, followed by preoccupied and dismissing styles, and the lowest scores in those with secure attachment (p = 0.003). After adjusting for covariates, fearful attachment remained an independent predictor of higher s-IAT-sex scores (B = 4.32; 95% CI [3.38–5.25]; p < 0.001).

**Conclusions:**

This study, the first in Tunisia on this topic, highlights the link between attachment style and sexual cyberaddiction. These findings emphasize the need for targeted interventions that address insecure attachment styles.

**Disclosure of Interest:**

None Declared

